# MGnify: the microbiome analysis resource in 2020

**DOI:** 10.1093/nar/gkz1035

**Published:** 2019-11-07

**Authors:** Alex L Mitchell, Alexandre Almeida, Martin Beracochea, Miguel Boland, Josephine Burgin, Guy Cochrane, Michael R Crusoe, Varsha Kale, Simon C Potter, Lorna J Richardson, Ekaterina Sakharova, Maxim Scheremetjew, Anton Korobeynikov, Alex Shlemov, Olga Kunyavskaya, Alla Lapidus, Robert D Finn

**Affiliations:** 1 European Molecular Biology Laboratory, European Bioinformatics Institute (EMBL-EBI), Wellcome Genome Campus, Hinxton, Cambridge CB10 1SD, UK; 2 Wellcome Sanger Institute, Wellcome Genome Campus, Hinxton, Cambridge CB10 1SA, UK; 3 Common Workflow Language, a project of the Software Freedom Conservancy, Inc. 137 Montague Street, Suite 380, Brooklyn, NY 11201-3548, USA; 4 Center for Algorithmic Biotechnologies, Saint Petersburg State University, Russia

## Abstract

MGnify (http://www.ebi.ac.uk/metagenomics) provides a free to use platform for the assembly, analysis and archiving of microbiome data derived from sequencing microbial populations that are present in particular environments. Over the past 2 years, MGnify (formerly EBI Metagenomics) has more than doubled the number of publicly available analysed datasets held within the resource. Recently, an updated approach to data analysis has been unveiled (version 5.0), replacing the previous single pipeline with multiple analysis pipelines that are tailored according to the input data, and that are formally described using the Common Workflow Language, enabling greater provenance, reusability, and reproducibility. MGnify's new analysis pipelines offer additional approaches for taxonomic assertions based on ribosomal internal transcribed spacer regions (ITS1/2) and expanded protein functional annotations. Biochemical pathways and systems predictions have also been added for assembled contigs. MGnify's growing focus on the assembly of metagenomic data has also seen the number of datasets it has assembled and analysed increase six-fold. The non-redundant protein database constructed from the proteins encoded by these assemblies now exceeds 1 billion sequences. Meanwhile, a newly developed contig viewer provides fine-grained visualisation of the assembled contigs and their enriched annotations.

## INTRODUCTION

Microbiome research typically involves the study of the collective genetic material of microorganisms from a given environment (known as a biome). This diverse and expanding research field (in terms of breadth of biomes, methods and scientific questions) has been applied to a wide variety of environments, from the abyssal waters and sediments of the world's oceans (1–3), to ice and soil from the world's highest mountains (4,5), and almost every conceivable biome between (and even beyond (6)). This growth is also reflected by the number of datasets that can be found within the European Nucleotide Archive (ENA) (7). At the time of writing, over 1.9 million raw read microbiome datasets are publicly available, with 31.5% of these having been released in the last year.

As the field matures, microbiome analysis is increasingly redefining our understanding of microbiology by providing unique insights into microbial community composition, the processes performed by the microbes and their relationships with their surroundings and each other. For example, recent microbiome studies have identified a global core bacterial community in wastewater treatment plants that is strongly linked to activated sludge performance (8); have highlighted differences in the gut microbiota of babies delivered by caesarean-section compared to vaginal birth (9); and have discovered a number of microbial gene products that metabolize orally administered drugs, with potential effects upon medical therapy (10). Meanwhile, research efforts focused on human gut (one of the most extensively studied biomes to date) have recently revealed unprecedented numbers of novel bacterial species with new potential functions (11–13).

MGnify (previously known as EBI Metagenomics (14)) is a freely available hub for the analysis, exploration and archiving of microbiome data. The resource accepts user-submitted data and provides standardized pipelines that offer taxonomic and (where appropriate) functional analysis of microbiome datasets. Amongst the data types covered are studies that target taxonomic markers, such as the small subunit (SSU) ribosomal ribonucleic acid gene (amplicon studies), whole genome shotgun sequencing studies (metagenomics) and whole transcriptome shotgun sequencing studies (metatranscriptomics). More recently, the resource has begun to offer analysis of user-submitted assembled sequence data (assembly), and/or can provide assembly of user-submitted metagenomic data prior to analysis (available upon request).

Through partnership with the ENA, sequencing data and metadata submitted for analysis are accessioned and stored permanently within the archive, which operates under the International Nucleotide Sequence Database Collaboration (INSDC) (15). This ensures efficient storage of data, which may be held under pre-publication status (typically for 2 years), and provides a permanent record of the raw sequence data. Meanwhile, the analyses of these datasets (both pre-release and publicly available) reside within MGnify, which provides an independent website and API for data discovery and exploration. In MGnify, analysis of user-submitted data is supplemented by processing publicly available microbiome datasets drawn from the INSDC via the ENA. Furthermore, MGnify users can request that any relevant publicly available study found within the INSDC can be analysed with the latest MGnify pipeline version, and the results added to the resource. Enabling large scale data analysis using standardized pipelines in this way allows studies to be placed in context with each other, increasing data reuse and maximizing the knowledge that can be extracted from the datasets.

Here we report a number of major developments within MGnify since our last update (14), including substantial growth of the resource and development of new analysis components that expand the available annotations, facilitating greater insights into microbiome composition and function.

## UPDATES TO MGnify CONTENT

At the time of the renaming of resource in June 2018, unique and stable accession numbers for studies and analyses in MGnify were introduced, replacing the study and run accessions, respectively, that were inherited from INSDC. Driven by ongoing efforts to adhere to the FAIR data principles (16) (aimed to make data findable, accessible, interoperable and reusable), this change allows users to distinguish analysed microbiome datasets from the primary sequence data in ENA - which continue to be presented under the study and run accessions - and helps identify where MGnify adds content.

Analysed studies in MGnify are assigned the accession prefix MGYS followed by eight digits, and are linked to the corresponding sequencing project in the ENA. The distinction between MGnify studies and ENA projects is important, as MGnify curators add supplementary metadata to imported studies, assigning a relevant biome selected from the Genomes OnLine Database (GOLD) (17) biosample hierarchy. MGnify study metadata derived from ENA is also checked and corrected where necessary (e.g. correcting inverted latitude and longitude values). MGnify studies also have links to the corresponding ENA samples and runs (i.e. where the full metadata and source sequence data can be found). Analyses performed on the sequencing runs are assigned accessions, which are prefixed with MGYA followed by eight digits.

In the ENA, projects, samples and runs are arranged in one-to-many relationships, where one project may contain several samples that can each have a number of associated runs (e.g. technical replicates or different experiments performed upon the same sample material). The same structure is used in MGnify, with additional one-to-many relationships between the individual sequencing runs and their analyses, as one run file may be analysed with multiple pipeline versions. In some cases, it is desirable to group together multiple related studies that may have been submitted at different times by different research groups or sequencing centres, but form part of a larger overarching project. To enable this, we have added the concept of ‘Super Studies’ to the MGnify data model, which allows the grouping of related projects under a single entry point. The generation of the Super Studies groupings is currently community driven (via requests to the MGnify helpdesk), and the groupings are expected to expand over time. The list of current ‘Super Studies’ can be accessed in the MGnify website via a menu under the ‘Browse data’ tab. For example, at present, all 96 individual studies that make up the Earth Microbiome Project (18) are grouped together under a single Super Study, as are MGnify analyses for 8 distinct Tara Oceans (19) datasets. The Super Studies facility makes it easier to discover, explore and compare data across such large-scale projects.

In terms of overall data, MGnify currently contains over 3500 publicly available projects, comprising ∼175 000 samples, ∼230 000 runs and ∼240 000 analyses. This represents a 2-fold increase in the number of datasets analysed over the last two years. The majority of this data (∼193 000 analyses) are 16S or 18S rRNA gene amplicon datasets, followed by metagenomic (∼27 000) and assembly (∼16 000) analyses, with a smaller number of analysed metatranscriptomes (∼2000) and metabarcoding runs (∼2000, the majority of which target internally transcribed spacer (ITS) regions - as previous pipelines could not analyse ITS regions, these datasets will be prioritised for analysis with the new pipeline, described below). The different proportions of analyses (amplicon, metagenomics and assembled) closely reflect the corresponding proportions of raw sequence data found in ENA.

Whilst analysis of raw reads can provide detailed insights into the microbiome, assembly of the reads into longer contigs can underpin a deeper understanding of the data through recovery of full-length proteins, prediction of complete biochemical pathways and/or biological systems, and potential reconstruction of complete genomes. In addition to the analysis of data, MGnify is engaged in a parallel exercise, assembling publicly available metagenomic datasets from ENA. To date, we have assembled ∼43 000 sequencing runs drawn from a wide range of biomes, and are in the process of submitting the assembled contigs back into the ENA. Approximately one third of these assemblies have been uploaded and analysed with MGnify to date (accessible via https://www.ebi.ac.uk/metagenomics/search#analyses, and selecting the ‘Assembly’ facet found under the ‘Experiment Type’ section of facets, listed on the left of the page). Analysis of the remainder, plus additional assembled datasets, will be a priority over the coming years.

## ASSEMBLY OF SHOTGUN METAGENOMICS DATASETS

Since assembly of shotgun metagenomics data was introduced to MGnify in 2018, we have seen a high uptake of this new feature, both in terms of public datasets being requested for analysis and for private analysis. Assembly is a computationally expensive process, frequently requiring terabytes of memory to accomplish when using metaSPAdes (20), which is our primary assembly algorithm, as the ensuing assemblies are typically of better quality than those produced using other assemblers (21). Indeed, a similar conclusion has been drawn by the IMG/M resource (22), who are currently backfilling old assemblies that used an alternative approach with metaSPAdes derived assemblies. Over the past two years, only a handful of datasets (<0.1%) out of the ∼43 000 assemblies have not been produced using metaSPAdes, due to memory requirements exceeding the maximum available. We are also currently working with the metaSPAdes developers on strategies to predict memory utilisation (*a priori*) and ways to decompose the metaSPAdes algorithm to maximize the resource utilizations (in other words, only occupy the high memory compute nodes when absolutely necessary and/or to utilize multiple nodes to virtualize a high memory node).

Our ambition is to ensure that all of the assemblies generated by MGnify are submitted into the ENA, in line with our data policy that all sequence datasets we analyse must be appropriately archived within INSDC. Public datasets are unproblematic, as we (MGnify) submit them as ‘Third Party datasets’ in accordance with the ENA submission policy. However, assembled private datasets can not be brokered into the ENA by MGnify on behalf of the submitter. Thus, we currently require the user to submit assemblies under their account credentials, in order for them to maintain ownership. This presents an additional burden for the user, where we assemble their raw sequence data and then pass it back to them for submission before it can be analysed (a situation we are working to resolve). However, this does enable many users who lack the necessary computational infrastructure or expertise to access metagenomics assembly.

## PIPELINE UPDATES

Our previous analysis pipeline (v4.1) was released in January 2018 and added a range of new features, including taxonomic profiling of eukaryotes based on small and large subunit ribosomal ribonucleic acid (SSU and LSU) sequences, and support for assemblies. The latest update (v5.0), released September 2019, extends the analyses further, substantially increasing the scope of functional annotations through the addition of KEGG (23) orthologue predictions for reads and assembled contigs, and KEGG pathways and Genome Properties (24) annotations for assembled contigs. The latter two represent higher order annotations (based on two or more proteins) that enable the description of functional systems encoded by the microbes within a metagenome.

## IMPLEMENTATION OF THE PIPELINE

Since the first inception of the analysis pipeline in 2011, there have been five major versions. Each version has included both updates to reference databases and incremental expansion to the range of analyses provided to the user. With increasing complexity of analyses and growing numbers of tools, reference databases and input parameters, clearly communicating all aspects of the pipeline (thereby providing both provenance and reproducibility) has become a non-trivial problem. This issue has been highlighted by community papers (25) and the Genomics Standard Consortium, which has a specific working group dedicated to metagenomics analysis. With this in mind, our recent previous pipeline versions (4.0 and 4.1) were accompanied by formal descriptions of the pipeline using the Common Workflow Language (CWL) standards (https://www.commonwl.org/). However, for these pipeline versions, the CWL documents were retrofitted to match our internal software stack. This software stack lacks portability as it was designed for our internal compute infrastructure and combines data processing functions, software parameters and execution logic. The new pipeline is a major departure from this practice, where we have defined the CWL workflow first, and now utilize toil-cwl-runner (a CWL compatibility layer that is a part of the Toil workflow execution engine (26)) to orchestrate the execution on our compute infrastructure. This has removed the duplication of effort (in terms of implementing the logic in software and then retrospectively producing a CWL description), and helps ensure provenance, interoperability and reproducibility of the pipeline.

To enable the new range of analyses, we have also departed from our one-size-fits-all single pipeline approach, and introduced analysis based on the input data type (namely amplicon, metagenomic/metatranscriptomic raw reads and assemblies). While this adds complexity to the triggering of the appropriate analysis pipeline, the CWL v1.x standards avoids conditional branching to simplify execution, meaning this has been a necessary change. Nevertheless, as the overarching CWL workflow is built from various modules or sub-workflows, it becomes relatively simple to compare the overlap between the different workflows using visualisation tools such as the CWLViewer (https://view.commonwl.org). Below we outline the three new analysis pipelines, highlighting the extended analysis functionality that has been included between versions 4.1 and 5.0. Within the downloads sections of each analysis, we now provide the appropriate link to the CWL workflow used and the associated configuration file, providing the full provenance of the analysis (see: https://github.com/EBI-Metagenomics/pipeline-v5, and schematically presented in the Supplementary Figures S1–S3).

## AMPLICON PIPELINE

In version 5.0 of the pipeline, analysis of amplicon datasets has been expanded from SSU and LSU-based classifications to include those based on ITS regions. These regions lie between the SSU and LSU genes and can be targeted for sequencing to provide taxonomic classification of eukaryotic organisms, particularly fungi. As with version 4.1 of the pipeline, for amplicon analysis, paired end sequences are merged with SeqPrep (v1.2) (27) and undergo quality control (QC), with low quality sequencing regions trimmed using Trimmomatic (0.36) (28) and sequences shorter than 100 nucleotides in length removed. Since the target of an amplicon study may be mixed (e.g. the study may contain both 16S rRNA gene and ITS analyses) or unspecified, all amplicon datasets are compared against the SSU and LSU models from Rfam (v13.0) (29), with analysis performed on the matching sequences using MAPseq (v1.2.3) (30) in conjunction with the SILVA (v132) database (31). Additional ITS-based classification is performed as follows: SSU and LSU regions are masked in the sequence set and ITS analysis is carried out using MAPseq and two separate databases - ITSoneDB (v1.138) (32) and UNITE (v8.0) (33).

The two ITS reference databases offer complementary coverage. ITSoneDB is a collection of eukaryotic ITS1 sequences with a broad taxonomic range. UNITE, meanwhile, contains both ITS1 and ITS2 sequences and is widely used by the scientific community (for example, UNITE is used within the Global Biodiversity Information Facility: https://www.gbif.org), but is more focused on fungal sequences. In order to make them compatible with MAPseq, the ITSoneDB and UNITE reference databases were remapped to an eight-level taxonomy, using in-house scripts. To minimize cross-reactivity against sequences that may contain SSU and LSU regions, both databases were screened against the Rfam SSU and LSU rRNA models and matching sequence regions were masked.

**Figure 1.**
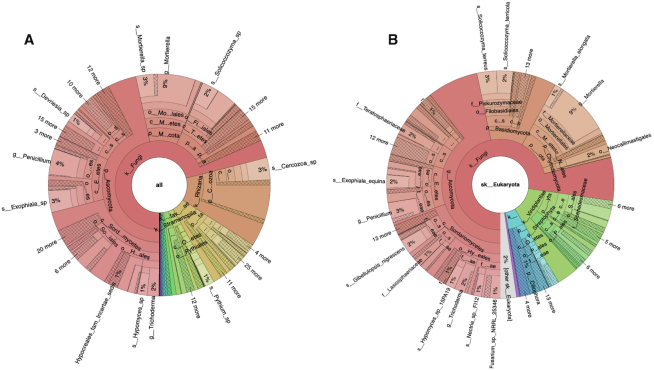
Krona plots showing ITS-based taxonomic analysis results for sequencing run ERR2237853 from agricultural soil. Panel A shows the analysis results obtained using the UNITE database and B shows those produced with ITSoneDB. These plots are integrated into the taxonomy tab within the analysis results section, https://www.ebi.ac.uk/metagenomics/analyses/MGYA00383253#taxonomic.

The outputs of the amplicon pipeline, whether SSU-, LSU- or ITS-based, can be visualised on the website as Krona plots (34) (see Figure 1), bar charts and tables. The outputs, including the full MAPseq analysis results, can be downloaded from the website or accessed via the API. The overall results for all runs analysed within a study are summarized as matrix files and are available under the ‘Analysis summary’ tab on the Study page of the website.

## METAGENOMIC AND METATRANSCRIPTOMIC RAW READS PIPELINE

Both taxonomic and functional analyses of metagenomic and metatranscriptomic raw reads have been extended in the latest version of the pipeline. Following initial paired-end merging of sequences (where appropriate) and QC steps (as described above for amplicon datasets), Rfam is used to identify SSU and LSU sequences and other non-coding RNAs (ncRNAs). As with the previous version of the pipeline, taxonomic classification is then performed on the SSU and LSU sequences using MAPseq and SILVA, via a CWL sub-workflow in common with the amplicon pipeline. Additional phylogenetic marker gene-based operational taxonomic unit profiling is performed using mOTUs2 (35) on all reads that pass QC, which allows sensitive and accurate quantification for both known and unknown species.

Following masking of the sequences encoding RNAs, protein coding sequences are predicted using FragGeneScan (v1.20) (36). In common with the previous version of the pipeline, functional analysis of predicted protein coding sequences is performed using InterPro (37) (which has been updated to version 75.0) and Gene Ontology (GO) terms (38), the latter being summarized for visualization via a specialized GO Slim developed for metagenomic data (available at http://www.geneontology.org/ontology/subsets/goslim_metagenomics.obo). While previously available as part of the InterPro results, Pfam (39) annotations are now provided as an additional results set to aid comparison with other resources that use Pfam for annotation, such as IMG/M. Additional functional analyses are also provided in the form of KEGG orthologue (KO) (23) annotations, which are calculated using HMMER (40) and the KOfam library (using a slightly modified form of the profile hidden Markov model (HMM) database of KEGG orthologues (41)) on the predicted protein sequences. The full set of annotations can be visualized on the website as a series of graphical plots and tables. The data is also available via the API and summarized for the whole study as a series of matrix files.

## ASSEMBLY PIPELINE

**Figure 2.**
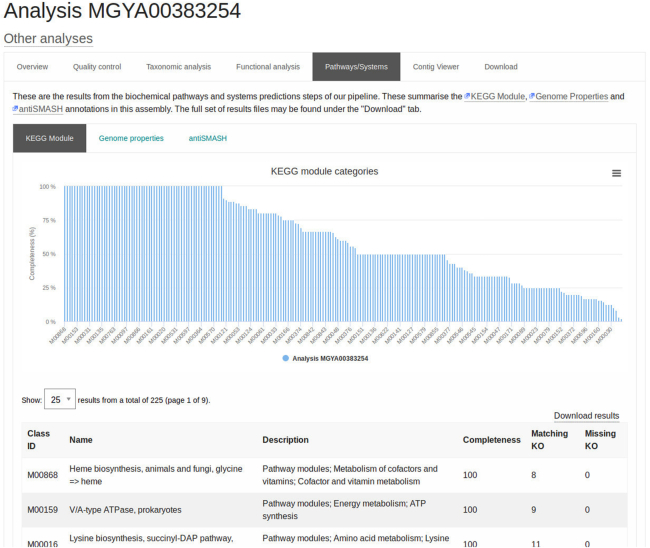
Screenshot showing the percentage completion for a series of KEGG pathways based on the presence of KEGG orthologues, https://www.ebi.ac.uk/metagenomics/analyses/MGYA00383254#path-systems.

Building on the extended functional analysis for raw reads, outlined above, a number of pathway and system annotations have been added for assembled contigs. Following filtering to remove contigs shorter than 500 nucleotides in length, SSU/LSU-based taxonomic predictions are run and RNAs are masked, as described for the raw reads analysis above. Protein coding sequences are identified through a combined gene caller that uses Prodigal (42) supplemented by running FragGeneScan on any sequence regions for which no proteins are predicted. The resultant protein sequences undergo functional predictions as described for raw reads, producing KO, InterPro, Pfam and GO term annotations. In addition, eggNOG (v4.5.1) (43) annotations are generated using the eggNOG-mapper tool (v1.0.3) (44). The KO results are also used to generate KEGG pathway annotations, as follows: network graphs are created for all KEGG pathways, where reactions are the nodes and KOs are the edges; based on the KO predictions for the proteins, in-house scripts are then used to iterate through all possible paths, and percentage pathway completion is calculated (as illustrated in Figure 2). In addition, InterPro annotations are processed to generate a compendium of Genome Properties results, detailing whether a property is present, partially present or absent in the dataset. antiSMASH (45) is also run on the predicted protein set, providing annotation of biosynthetic gene clusters. Finally, the proteins are also compared individually against the UniRef90 (46) database using DIAMOND (47) in ‘blastP’ mode to identify the accession, description and taxonomic identifier of the best matching sequence.

As well as being available to download from the website and API, annotations on the contigs can be visualized using a newly developed contig viewer, described below.

## ASSEMBLED CONTIGS VIEWER

Within the MGnify website, the majority of the analysis outputs are provided as tabular outputs and associated graphical plots. However, understanding the genomic context of proteins can provide greater insights into their functional roles. To enable users to access such information, the analysis section for assemblies now includes a ‘Contig Viewer’ tab that uses the Integrative Genomics Viewer (IGV) (48) framework to provide access to each contig with the corresponding functional annotations. As a metagenomics assembly may typically contain >1000 contigs, a range of parameters can be selected to filter the results. These include attributes such as contig length, coverage by raw reads and name. Additional filter parameters are based on the contig annotations, such as COG (49) category code (produced as part of the eggNOG annotations), KEGG orthologue accession, GO accession, Pfam accession and/or InterPro accession.

**Figure 3.**
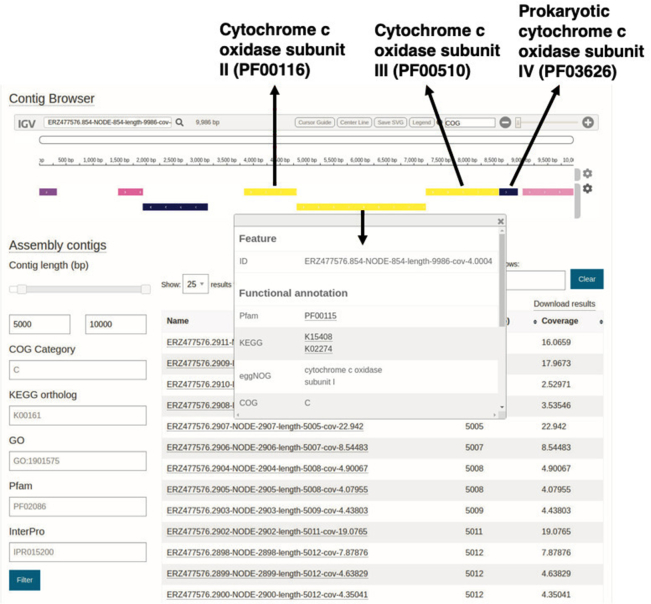
The MGnify contig viewer allows visualization of functional annotation of contigs, https://www.ebi.ac.uk/metagenomics/analyses/MGYA00383254#contigs-viewer. In this particular view, the contig ERZ477576.854-NODE-854-length-9986-cov-4.0004:1-9986 has been selected and the proteins coloured according to the COG category, with the four cytochrome *c* subunits highlighted.

Once contigs have been selected, annotations for the predicted proteins can be coloured according to their functional annotations (InterPro, GO, Pfam, EggNOG, COG and KEGG). Clicking on protein reveals a feature table of all annotations for that protein. Figure 3 shows an example of the contig viewer, with an example contig from assembly ERZ477576 (analysis MGYA00383254 of the assembled Tara Ocean study MGYS00002008), which contains the four subunits of the bacterial cytochrome oxidase.

## UPDATES TO THE MGnify PROTEIN DATABASE

Metagenomics has started to provide the scientific community with access to the ∼99% of organisms that are yet to be cultured (50). To allow users to explore the ever-expanding functional repertoire that metagenomics is revealing, MGnify produces a non-redundant protein database that is generated from amalgamating all open reading frame predictions from the analysis of all of the underlying assembled datasets. Over the past 2 years, this database has grown from 50 million sequences (v2017_08) to over 1.1 billion (v2019_05). As part of the MGnify renaming in 2018, each unique sequence was assigned a stable accession in the format ‘MGYP’ followed by 12 digits. Approximately 25% of these sequences are predicted to be full length according to Prodigal's gene identification criteria, with 15% truncated at either the N or C terminus and 60% predicted to be truncated at both ends (termed ‘partials’). Despite the substantial increase in number, there remains very little overlap with the sequences found in MGnify and those in UniProtKB, with only 9 million identical sequences in common between the two resources.

To better understand the redundancy within the MGnify protein database, and to provide a more manageable dataset for users, we have used Linclust from the MMseqs (51) suite to cluster the sequences using 90% thresholds for overlap (of the shortest sequence) and sequence identity. Due to the clustering parameters, the selected cluster representative (the centroid sequence), is typically the longest. Providing online sequence similarity searches against the entire protein dataset has not been possible, due to the size of the full database. However, we do provide the cluster representatives as a target database that can be searched online. A user can further restrict the search via the web interface, limiting searches to sequences originating from a particular subset of biomes, or according to the Prodigal prediction (full length, truncated or partial).

The entire set of protein sequences is available via FTP & HTTP (http://ftp.ebi.ac.uk/pub/databases/metagenomics/peptide_database/current_release/). In the most recent release of the protein database (v2019_05), each protein entry is mapped to the MGnify assembly or assemblies from which it is derived. Furthermore, we also provide the fasta headers of the sequences as given by Prodigal, providing a direct link to the contig in the assembly (identified by ENA ERZ accession), along with the coordinates of the gene.

## DISCUSSION

MGnify is one of the world's largest resources for the analysis of microbiome datasets. The updates described here close many of the gaps in its analysis repertoire, through the expanded scope of taxonomic and functional analyses, the range of data products and the visualizations provided by the website. Notably, these have particularly improved the interpretation of metagenomics assemblies. The expanded analyses have involved the incorporation of well-established tools and databases (such as eggNOG), as well as the introduction of more recently developed/updated tools (e.g. KOfams and mOTUs). In an effort to enable transparency of the analysis pipeline, particularly in terms of the tools (and their versions), parameters and reference databases that we employ, we have migrated to a formal description of the pipeline using the CWL standards. The adoption of third party workflow execution engines reduces the burden for maintaining pipelining software, and will enable us to take advantage of additional developments, such as CWLProv (52) that will formally capture additional provenance metrics (e.g. intermediate file checksums), corroborate the CWL and record resource utilisation (such as the CPU time and memory usage of each tool). The use of CWL also increases our ability to deploy the pipeline in different cloud computing platforms, although the containerization of all of the tools used within our pipeline that is necessary for this remains an ongoing effort. Such approaches are imperative to enable us to continue to scale the MGnify analysis and assembly services to meet the ever-growing volume of data and demand for them.

We continue to monitor the field, in terms of identifying new tools, expanding focus areas and increasing use of different types of sequencing technologies. Based on our current observations, we are developing additional pipelines to provide greater support for long read sequencing technologies. To our knowledge, MGnify and IMG/M are the only major resources offering metagenomic assembly as a service, with both having large, inequivalent collections of metagenomics assemblies. While assembly of large shotgun metagenomics datasets remains computationally expensive, the assembled data (potentially) provides a deeper understanding of microbiomes, providing access to full length proteins, enabling the assertion of pathways and systems, and are foundational to the reconstruction of genomes (i.e. metagenome assembled genomes, known as ‘MAGs’). As MGnify develops further over time, we expect to continue to maximize the knowledge that can be gleaned from our assemblies, increasing support for generating and displaying MAGs, tightening the integration between the MGnify protein dataset and the corresponding contigs from which they are derived and including viral taxonomic annotations based on the contigs.

## Supplementary Material

gkz1035_Supplemental_File
