# Supplementary material for: MGnify: the microbiome analysis resource in 2020
Source: Nucleic Acids Res. 2019 Nov 7;48(D1):D570–8. doi: 10.1093/nar/gkz1035 (PMC7145632; doi:10.1093/nar/gkz1035)
Supplement: gkz1035_Supplemental_File [file gkz1035_supplemental_file.docx]

Supplementary Figures


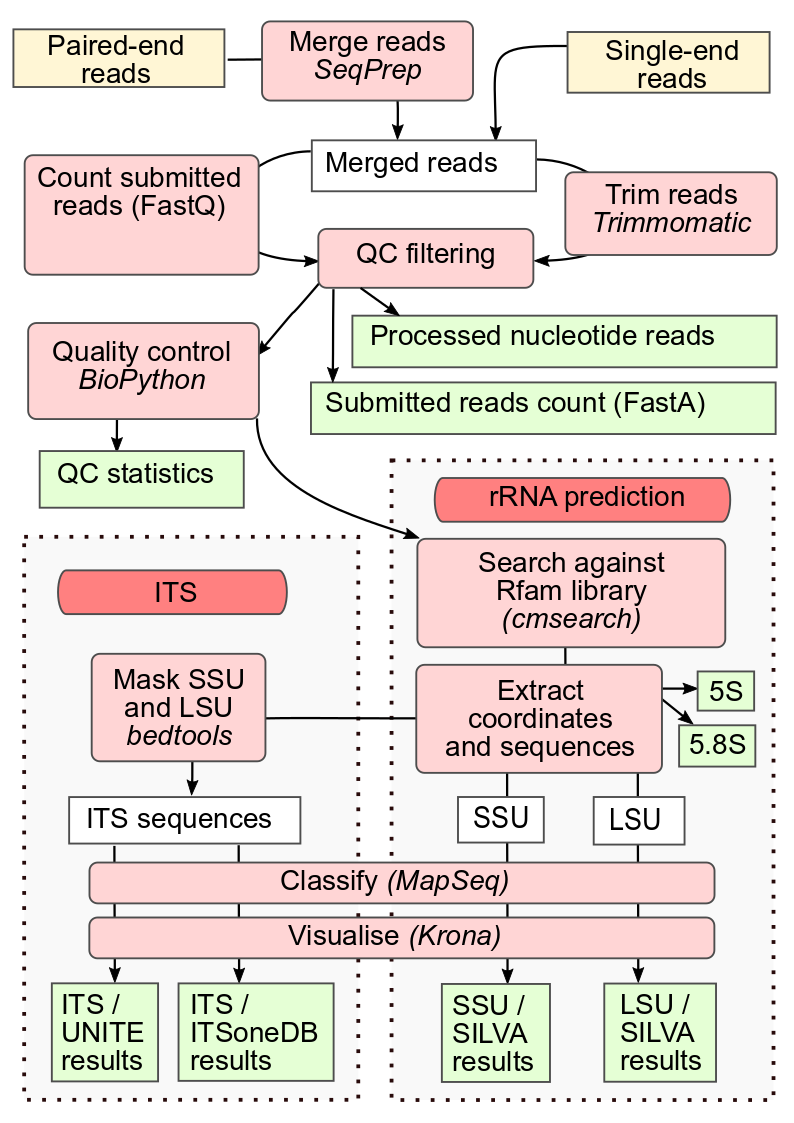


Supplementary Figure 1 – Schematic of the MGnify amplicon analysis pipeline.


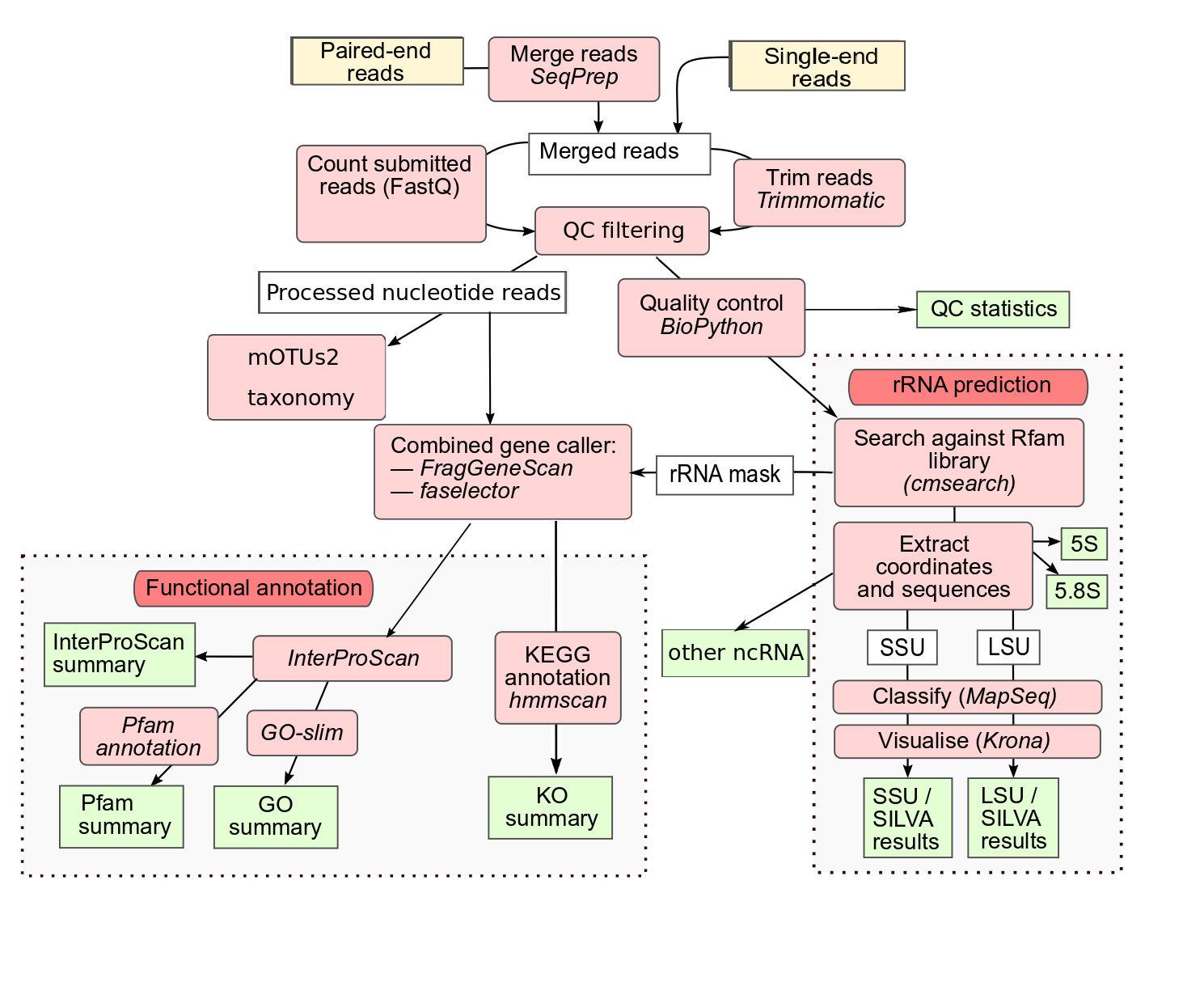


Supplementary Figure 2 – Schematic of the MGnify raw-read analysis pipeline.


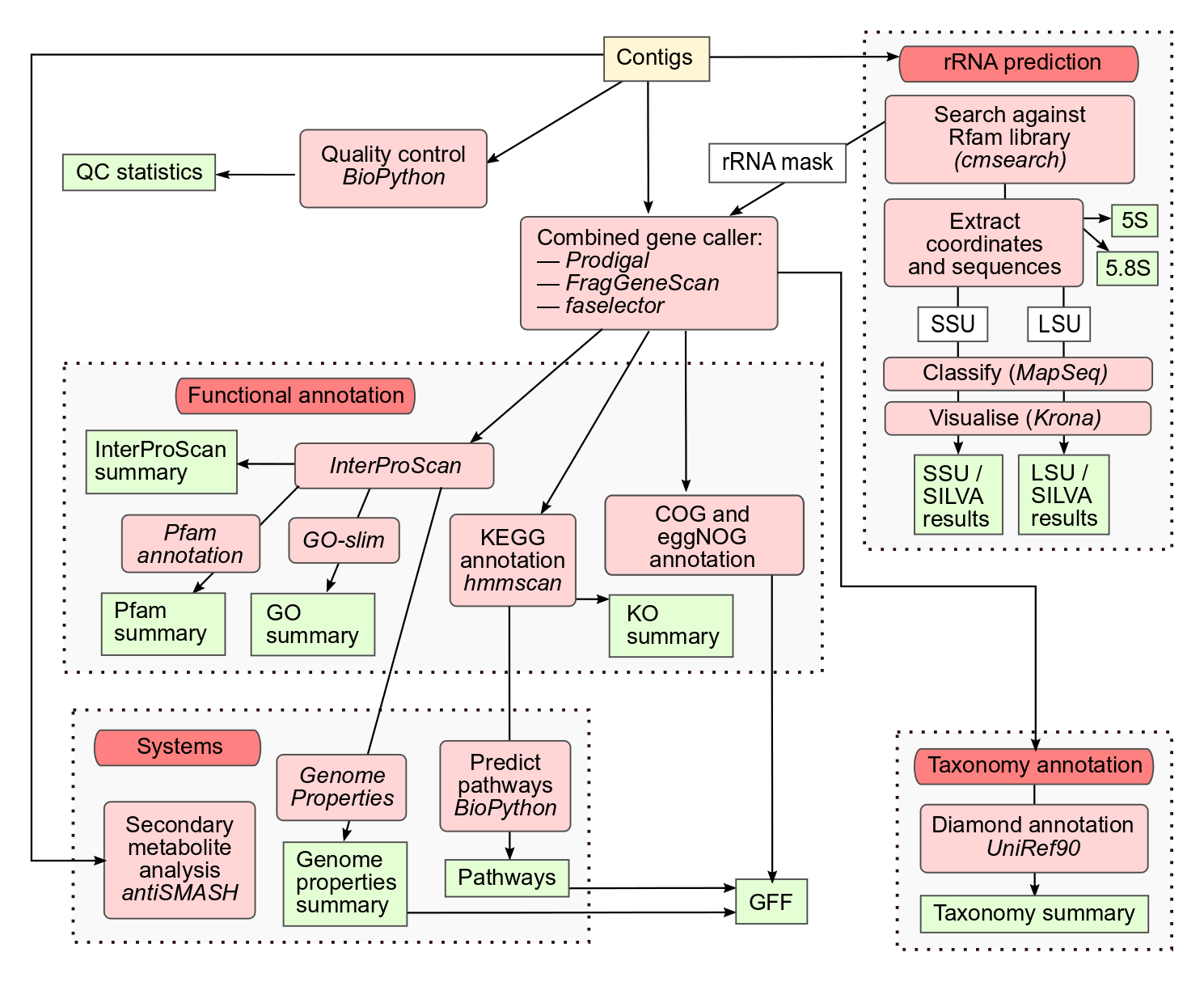


Supplementary Figure 3 – Schematic of the MGnify assembly analysis pipeline.
